# Web-based guided self-help for employees with depressive symptoms (Happy@Work): design of a randomized controlled trial

**DOI:** 10.1186/1471-244X-13-61

**Published:** 2013-02-18

**Authors:** Anna S Geraedts, Annet M Kleiboer, Noortje M Wiezer, Willem van Mechelen, Pim Cuijpers

**Affiliations:** 1 Department of Clinical Psychology, VU University, Amsterdam, The Netherlands; 2 EMGO Institute for Health and Care Research, VU University Amsterdam and VU University Medical Center, Amsterdam, The Netherlands; 3 Body@Work, Research Center Physical Activity, Work and Health, TNO-VU-VUmc, Amsterdam, The Netherlands; 4 TNO, Hoofddorp, The Netherlands; 5 Department of Public and Occupational Health, VU University Medical Center, Amsterdam, The Netherlands

**Keywords:** Internet, Depression, Absenteeism, Employees, Occupational therapy, Self-help, Cognitive therapy, Problem solving, Randomized trial.

## Abstract

**Background:**

Depressive disorders are highly prevalent in the working population and are associated with excessive costs for both society and companies. Effective treatment for employees with depressive symptoms in occupational health care is limited. The purpose of this study is to investigate the effectiveness and cost-effectiveness of an indicated preventive web-based guided self-help course for employees with depressive symptoms.

**Methods:**

The study is a two-arm randomized controlled trial comparing a web-based guided self-help course with care-as-usual. The self-help course consists of 6 weekly lessons. Weekly support will be provided by a coach via the website. Subjects in the care-as-usual group do not receive any treatment in addition to regular care. 200 white collar workers from several national and international companies in the Netherlands will be recruited via different methods such as banners on the company’s intranet, pamphlets and posters. Subjects will be included when they: have elevated depressive symptoms (score ≥16 on the Center for Epidemiologic Studies Depression scale), are 18 years of age or older, have access to the Internet and can be contacted via e-mail. Exclusion criteria are: partial or full work absenteeism, a legal labor dispute with the employer and receiving treatment from the company’s occupational health care at study entrance.

The primary outcome is depressive symptoms. Secondary outcomes include work absenteeism, work performance, burnout, anxiety, quality of life, health care use and production losses. Outcome data will be collected at 8 weeks, 6 months, and 12 months after baseline. Analyses will be based on the intention-to-treat principle. The cost-effectiveness analyses will be performed from a societal and a company’s perspective. A process evaluation will be conducted alongside the study.

**Discussion:**

This study evaluates the effectiveness and cost-effectiveness of a web-based guided self-help course for employees with depressive symptoms. This study could stimulate the use of e-mental health interventions in the worksite setting.

**Trial registration:**

Nederlands Trial Register (NTR): TC2993

## Background

### Depressive disorders and costs

Depressive disorders are highly prevalent in the general [1-3] and working [4,5] population and lead to excessive costs [6,7]. In the Netherlands, the lifetime prevalence of major depressive disorder is 15.4% [8,9] and high costs have been reported [10-14]. Smit et al. [11] calculated and compared the total costs of common mental disorders, such as mood disorders, anxiety disorders and alcohol disorders in a Dutch population-based cohort study. Mood disorders showed the highest total costs of all common mental disorders. The per capita annual excess costs for a mood disorder were €5009, which equals costs of €311 million per one million persons aged 18–65 years. A substantial part of the total costs of depressive disorders are due to work absenteeism, work impairment, and loss of work productivity [11,15-21] with estimates ranging between 70-85% [11,12,15]. In a recent Dutch cohort study [10], the annual total costs for work absenteeism due to depressive disorders were calculated and estimated at €242 million per 1 million workers, which equals €1.8 billion for the entire Dutch working population. These costs are directly paid for by companies.

### Treatment of depressive disorders in the general population and the workplace

In the past twenty years, research on effective treatments for depression has been extensive [22-25]. Several meta-analyses have shown that depressive disorders can be treated effectively with pharmacotherapy, psychotherapy, or a combination of pharmacotherapy and psychotherapy [22-24]. More recently, the number of studies examining the effect of self-help treatment for mental disorders has increased substantially, especially for studies examining the effect on depressive disorders [26-28]. In addition, self-help delivered via the Internet with guidance of a professional counsellor has become available. Research has shown that the effect of this type of treatment is promising [27,29,30].

Compared to treatments for depression in general healthcare, the evidence for effective worker-directed treatments of employees with depressive symptoms is scarce [31]. Considering the importance of work-related factors in the development and perpetuation of depression, it may be important to involve the employability, disability in job performance and the work setting of the patient in depression treatment [31]. Treatment of employees with depressive symptoms can be either carried out in the workplace or in general healthcare.

In the Netherlands, the standard care for sick-listed employees with depressive symptoms in the workplace is treatment by an occupational physician. An evidence-based guideline for treatment of psychological problems in general is available for occupational physicians. However, this guideline does not focus on depression specifically [32]. In recent years, some research has been done on effective treatment of sick-listed employees with mental health problems; however, results are conflicting [33-36]. Yet, there is hardly any evidence on effective treatments of depressive symptoms for employees who are not on sick leave [37]. Recently, Lexis et al. [37] showed positive results of a problem-solving treatment for employees with a high risk for sick leave due to depressive symptoms. However, in this study the severity of the depressive symptoms was low and the number of treatment sessions with a psychologist varied. Gärtner et al. [38] have started a study on the effect of a Workers’ Health Surveillance mental module for health service workers with mental health complaints. The goal of the module is to improve employees’ mental health and work functioning. Results of the study are not published yet. To our knowledge, these are the only studies in which the effect of a treatment for employees with mental health complaints who are not on sick leave is tested.

### Study objective

Companies pay a substantial part of the costs for depressive disorders due to work absenteeism and loss of work productivity [11]. An indicated preventive strategy to reduce mild to moderate depressive symptoms in employees who are not (yet) on sick leave could lower the costs for companies by reducing work absenteeism and loss of work productivity [16]. A guided self-help treatment might be a valuable and cost efficient strategy to treat employees with mild to moderate depressive symptoms. Therefore, we developed a web-based guided self-help course for employees with depressive symptoms who are not on sick leave. The aim of this study is to examine the effectiveness and cost-effectiveness of the self-help course called Happy@Work. In this paper we describe the design of this randomized controlled trial.

## Methods

### Study design

**Figure 1:**
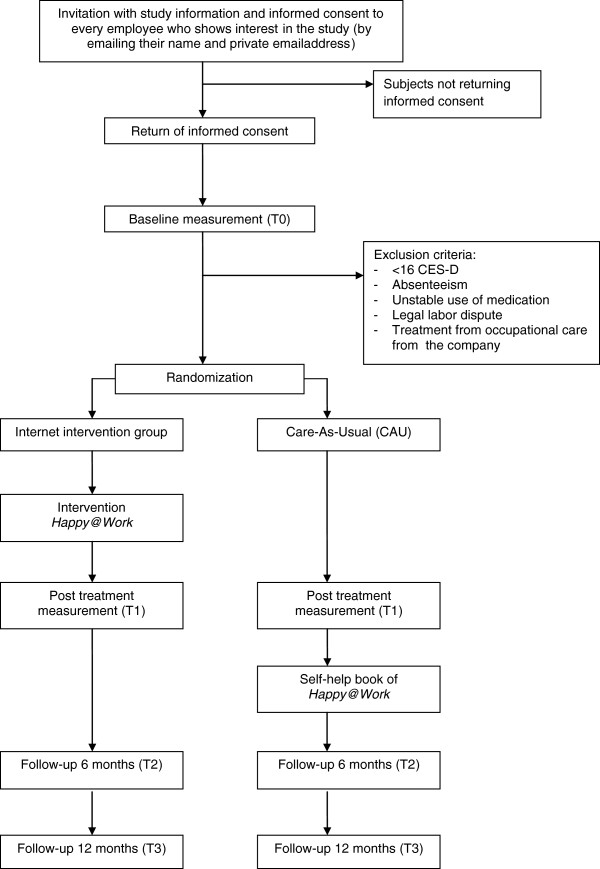
Overview of study procedure.

This study is a randomized controlled trial. Subjects will be randomized into two groups: an Internet guided self-help course or care-as-usual. The study protocol, information leaflet, and informed consent form were approved by the Medical Ethics Committee of the VU University Medical Center (registration number 2011/2). We use the CONSORT- EHEALTH guideline for reporting randomized controlled trials concerning ehealth [39]. The study design is shown in Figure 1.

### Inclusion and exclusion criteria

Employees with an increased level of depressive symptoms from companies with white collar workers (i.e. people who work in an office environment) in the Netherlands who are willing to enroll in a web-based guided self-help course are eligible for taking part in this study. Inclusion criteria are: 18 years of age or older, elevated depressive symptoms (scoring 16 or higher on the Center for Epidemiologic Studies Depression – scale [CES-D]), access to the Internet and an e-mail address. Exclusion criteria are: partial or full work absenteeism, receiving treatment from the company’s occupational health care at study entrance, unstable (<1 month) medication use for depressive symptoms, and having a legal labor dispute with the employer.

### Recruitment

Subjects will be recruited via different methods such as banners on the company’s intranet, pamphlets and posters. Subjects who are interested in taking part in the study can ask for more information about the study via e-mail. When information is requested, one of the researchers will send an information leaflet and an informed consent form via email. The informed consent form can be returned via post or e-mail. Additionally, subjects will receive an e-mail with a link to an online screening questionnaire. Subjects who are eligible to take part will be included.

### Randomization

Randomization will take place at an individual level after completion of the baseline measurement (questionnaire and clinical interview). We will use stratification at two levels: 1. use of antidepressants and 2. receiving treatment from a psychologist or psychiatrist at study entrance. Block randomization will be used with random blocks containing 4, 6 or 8 allocations. An independent researcher will make the allocation schedule with a computerized random number generator and the investigators will be unknown to the schedule. Subjects will be randomized into two groups: the intervention group or the care-as-usual group. Subjects will be informed about the randomization outcome via e-mail. Subjects in the intervention group will usually start with the intervention on the first Monday after randomization.

### Intervention

#### Intervention group

The intervention *Happy@Work* is a brief web-based intervention with guidance. It is based on Problem Solving Treatment (PST) [40], Cognitive Therapy (CT) [41] and a guideline to help employees with stress symptoms [42,43]. We adapted the content of an evidence based web-based PST intervention called “Allesondercontrole” (i.e. “Everything Under Control”) [29] and added exercises and examples. Furthermore, we included information and exercises about automatic thoughts and a mood diary (CT). In PST, it is assumed that depressive symptoms can be caused by practical problems that people face in their daily lives. It is believed that, when people can resolve their problems, their symptoms of depression will decrease [29]. The PST will help them solve their problems. Sometimes, however, problem solving can be disrupted by automatic thoughts such as ‘’I am too weak to solve this problem” or “I will fail solving this problem”. PST may not be sufficient to change these automatic thoughts that disrupt problem solving. Therefore, we incorporated CT information and exercises to change these automatic thoughts in the course [41]. Some of the problems that people face are likely to be work-related. These problems are sometimes more difficult for people to comprehend [42,43]. Therefore, one lesson is focused on work-related problems specifically. All examples in the intervention apply to white collar workers.

Participants will receive a username and password at the beginning of the course for secured and personal access to the website. Participants follow one lesson per week. Each lesson has a different theme, but always follows the same structure: information about the theme, examples, and assignments. When participants have finished the assignments, they will submit them via the website. A new lesson can be started after receiving the feedback from the coach (i.e. tunnelled intervention).

A coach will provide written weekly support via the website after a lesson has been completed. The support will be delivered by occupational social workers from the company or by Master level students in clinical psychology when occupational social workers are not available. The support includes feedback on the assignments, what went well and what can be improved. Additionally, we will also use motivational and empathic strategies to keep participants engaged in the course. Development of a patient-therapist alliance, as in traditional psychotherapy, is not an aim of the support.

The support will be placed on the website by the coach within three working days after the lesson was completed by the participant. All support will be reviewed by a supervising psychologist (usually the first author of this paper) to ensure the quality of the support. An automatic email will be send to participants once the support is placed on the website by the coach. The e-mail informs participants that support is provided, describes the theme of the next lesson, and contains the deadline for the next assignment. Email reminders will be sent to the participant when deadlines are not met.

The coaches will receive a six hour training consisting of education about problem solving therapy, explanation of the treatment manual which is used throughout the course, and practice with case material. The training will be provided by a supervising psychologist (usually the first author of this paper).

#### Care-as-usual group

Subjects in the care-as-usual (CAU) group will not receive treatment or support from the researchers. However, they are free to seek any help they want. In an email containing the randomization outcome they will be advised to consult their (occupational) physician or a psychologist if they want treatment for their depressive symptoms. Subjects who are interested in receiving the self-help book version of the intervention will receive a copy after they have completed the post-treatment assessment.

### Assessments

There will be a total of four assessments. The first assessment (baseline, T0) will be before the start of the intervention. Other assessments will be taken at 8 weeks (T1), 6 months (T2), and 12 months (T3) after baseline. At T0 and T2 a clinical interview, for diagnostic purposes, will take place via telephone. All assessments will be performed via online questionnaires from NetQuestionnaires (http://www.netq-enquete.nl/).

### Outcome measurements

#### Primary outcome

##### Depressive symptoms

Symptoms of depression will be measured with the Center for Epidemiological Studies Depression – scale (CES-D) [44]. This questionnaire is widely used for identifying people with depressive symptoms. Its validity has been tested in different populations [44-47]. The CES-D consists of 20 items and the total score varies between 0 and 60. A score of 16 or higher represents a clinically significant level of depressive symptoms [44]. The cut-off score of 16 will be used in this study as an inclusion criterion.

### Secondary outcomes

#### Work absenteeism

Work absence will be measured via self-report with the TiC-P (see *Costs: health care utilization and production loss*). Additionally, sick leave data from the companies will be used when available and can be merged into frequency and duration of sick leave during the time of the inclusion period of the study (12 months).

### Work performance

We will use the general work performance scale of the WHO Health and Work Performance Questionnaire (HPQ) [48] which contains four items. Subjects will score the general work performance of other colleagues, their own usual work performance during the last two years, their overall work performance during the past four weeks, and their overall work performance during the past four weeks when compared to their colleagues. Every item will be scored from 0 to 10, with higher scores indicating better work performance [49].

### Burnout symptoms

Burnout symptoms will be measured with the Dutch version of the Maslach Burnout Inventory-General Scale (MBI) [50], called the UBOS [51]. This self-report questionnaire contains 15 items and three dimensions: Exhaustion (5 items); Cynicism (4 items); Reduced professional efficacy (6 items). For every dimension a total score will be calculated. Participants with a high score on Exhaustion and a high score on Cynicism or a low score on Reduced professional efficacy will be considered as “burnout” [51].

### Anxiety symptoms

The anxiety subscale of the Hospital Anxiety and Depression Scale (HADS) will be used to measure anxiety symptoms [52]. The anxiety subscale of the HADS consists of 7 items. Scores range from 0 to 21 with higher scores indicating more anxiety. The HADS has shown good homogeneity and reliability in different normal and clinical Dutch samples [53].

### Quality of life

Quality of life will be assessed with the EQ-5D (EuroQol) [54]. The EQ-5D is a well validated instrument for measuring general health-related quality of life. It consists of 5 dimensions (mobility, self-care, usual activities, pain/discomfort, and anxiety/depression) which are rated as causing ‘no problems’, ‘some problems’ or ‘extreme problems’. The EQ-5D distinguishes 245 unique health states. Each unique health state can be assigned with a utility score which ranges from 0 (poor health) to 1 (perfect health). We will use such single EQ-5D summary index scores obtained in the Dutch population to calculate quality-adjusted life-years (QALYs) [55]. The EQ-5D also contains a Visual Analogue Scale (VAS) on which subjects rate their own health between 0 (worst imaginable health state) and 100 (best imaginable health state).

### Costs: health care utilization and production loss

A revised version of the Trimbos and iMTA Questionnaire on Costs Associated with Psychiatric Illness (TiC-P) [56] will be used to collect data on direct and indirect costs from the subjects. The TiC-P is a self-report questionnaire and consists of two different parts that can be administrated separately. Part I consists of 12 items and is concerned with measuring healthcare utilization by subjects. Questions on the frequency of utilization of health care services of the company such as occupational physician and social work were added to this part of the TiC-P.

Part II (Short Form Health and Labor Questionnaire [SF-HLQ]) is meant to determine lost productivity costs resulting from absenteeism (being absent from work because of illness) and presenteeism (being present at work while ill which may lead to reduced efficiency) and consists of 11 items.

The TIC-P can be assessed with different recall periods [56,57]. At all assessments we will use the time period between the current assessment and the previous assessment. For the baseline assessment a recall period of three months will be used.

### Clinical interview

The World Health Organization Composite International Diagnostic Interview (CIDI, version 2.1) [58] is a structured interview to assess psychiatric diagnosis defined in the Diagnostic and Statistical Manual of the American Psychiatric Association, 4^th^ edition (DSM-IV) [59]. For this study, two sections of the CIDI will be assessed: the depressive disorders and dysthymic disorder section, and the “other” anxiety disorders (social phobia, panic disorder, agoraphobia and generalized anxiety disorder) section. The assessment typically lasts 20–45 minutes, depending on the mental state of the subject. The CIDI will be conducted by trained interviewers via telephone at baseline (T0) and after 6 months (T2).

### Other outcomes

We will use mastery and social support in explorative analyses as possible mediators or moderators.

We expect that the effectiveness of the intervention Happy@Work will be depended on mastery. A previous study by Warmerdam et al. [60] on the effectiveness of “Allesondercontrole” has shown a mediating effect of mastery. Happy@Work is based on this intervention “Allesondercontrole.” We will also investigate the possible mediating or moderating effect of social support because increasing the social support is an important theme in Happy@Work. Furthermore, social support has been proven to be an important predictor of depression [61-63].

Furthermore, we will use a course evaluation questionnaire and a non-response questionnaire to gain insight in the use of the intervention.

### Mastery

The Pearlin Mastery Scale [64] contains 7 items and measures how much an individual perceives having control over things in his or her life. The total score ranges from 7 to 35. A high score (internal mastery) indicates that the subject generally feels in control of situations. A low score (external mastery) indicates that the subject generally has the feeling of being that things are out of his own control. This questionnaire has good psychometric properties [64].

### Social support and conflicts

Eight items concerning social support from coworkers and supervisors and one item concerning conflicts with the supervisor will be used. These items are from the Netherlands Working Conditions Survey [65]. The items are based on Karasek’s concepts of “Supervisory support” and “Co-worker support” from the Job Content Questionnaire (JCQ) [66], and were translated into Dutch by Houtman et al. [67].

### Course evaluation

We will use an Internet Intervention Evaluation Questionnaire [van Straten, unpublished data] to gain information from the intervention group about their experiences with the intervention. This questionnaire contains both quantitative (e.g. grades for website, feedback etc.) and qualitative (e.g. suggestions for improvement of the website, perceived effectiveness of the course etc.) questions.

### Non response

An adapted version of the Internet Intervention Adherence Measure [68] will be used to identify obstacles that interfere with the participant completing the Internet course. Obstacles are categorized as Internet/computer/technical issues, Personal/family issues, Intervention-general issues, and Intervention-specific issues. Participants will respond to the 29 items on a 3-point scale from 1 to 3, indicating whether that obstacle has had ‘no part’ , ‘a small part’, or ‘a major part’ in why they stopped using the Internet course. This questionnaire will be send to the participant when he reports to stop with the Internet course or when the participant will not show any activity on the website during a continuous period of three weeks.

### Other questions

We will add demographic variables, medication use for psychological problems and treatment by a mental health specialist to the questionnaire at baseline assessment.

For an overview of the outcome measurements see Table 1.

**Table 1:** Overview of outcome measurements

		**Time of measurement**			
**Instrument**	**Aim**	**T0 Baseline (pre-test)**	**T1 Post-test (8 weeks)**	**T2 Follow-up (6months)**	**T3 Follow-up (12 months)**
**CES-D**	Symptoms of depression	X	X	X	X
**Work absenteeism**	Self-report of absence from work and objective sick leave data	X	X	X	X
**Work performance**	General work performance in different time periods	X	X	X	X
**MBI/UBOS**	Burnout symptoms	X	X	X	X
**HADS**	Anxiety symptoms	X	X	X	X
**EQ5D**	Quality of life	X	X	X	X
**TiC-P**	Health care utilization and production losses	X	X	X	X
**CIDI**	Diagnostic interview	X		X	
**Mastery**	Perceived control	X	X	X	X
**Social support and conflicts**	Social support from coworkers and supervisor and conflicts with supervisor	X	X	X	X
**Course evaluation**	Satisfaction with Happy@Work (only intervention group)		X		
**Other questions**	Characteristics	X			

### Process evaluation

The process evaluation will be conducted alongside the study with use of the guideline of Steckler and Linnan [69]. In a process evaluation, Steckler and Linnan [69] advise to include several key process evaluation components. These components are: context, reach, dose delivered, dose received, fidelity, implementation, and recruitment. We will add perceived effectiveness as another key component.

Data for the process evaluation will be obtained via the course evaluation questionnaire at T1. Data from the non-response questionnaire will be used to assess reasons for not completing the Internet course. Furthermore, we will use information from the website to determine compliance, percentage of participants completing the course, and to gain information on the number of lessons participants have completed. If possible we will evaluate the coaches’ experiences with the website, giving feedback, and preferences for use of the website in the future.

### Sample size

The sample size of this study is based on the expected difference on the primary outcome variable (i.e. depressive symptoms) between the intervention group and the CAU group. Based on a power of 0.80, an alpha of 0.05, and an expected drop-out percentage of 30%, we will need 100 subjects in each condition to show an effect-size of 0.50 (based on previous research [29,30]). Therefore, the total sample size is determined at 200.

### Statistical analysis

Analyses will be conducted according to the intention-to-treat principle (ITT). Handling of missing data will be determined after the inclusion period of the study.

To examine differences between the intervention group and the CAU group we will use regression analyses. A multilevel analytic approach will be used if non-independence of observations is an issue in the data and will be determined by calculating the Intra Class Correlations (ICC). We will also use Cohens’ *d* to measure the size of the effect. Effect sizes of 0.8 are assumed to be large; effect sizes of 0.5 are moderate; and effect sizes of 0.2 are assumed to be small [70].

Clinical significant change will be determined with norms for the primary outcome measure and with the Reliable Change Index [44,71]. We will use the cut-off score of 16 on the CES-D as an indication of recovery. Reliable Change will be used as an index for improvement.

The explorative analyses of the possible mediating and moderating effects of mastery and social support will be tested as suggested by MacKinnon [72].

### Economic analyses

The economic evaluation will be performed from both the societal and company’s perspective.

Costs typically have a highly skewed distribution. Policy makers need information on the difference in mean total costs between the intervention group and the CAU group to be able to estimate the total health care budget needed for a specific condition [73]. Therefore, bias-corrected and accelerated bootstrapping with 5000 replications will be used to estimate 95% confidence intervals around the mean difference in total costs between the treatment groups.

Incremental cost-effectiveness ratios (ICERs) will be calculated by dividing the difference in mean total costs between the intervention group and the CAU group by the difference in mean effects between the groups. The ICER is expressed in terms of additional costs per clinically significant change in depressive symptoms severity (cost-effectiveness analysis) and in terms of Quality Adjusted Life Years (QALY) (cost-utility analysis). Bootstrapping will be used to estimate the uncertainty surrounding the ICERs which will be graphically presented on cost-effectiveness planes. Cost-effectiveness acceptability curves and net monetary benefits will also be calculated. Cost-effectiveness acceptability curves show the probability that the intervention is cost-effective in comparison with usual care for a range of different ceiling ratios thereby showing decision uncertainty [74]. We will also perform a cost-benefit analysis from the company’s perspective in which the costs of the intervention will be compared to benefits in absenteeism, depression and work performance.

## Discussion

In this randomized controlled trial we will compare a group which will follow an indicated preventive web-based guided self-help course for non-sick-listed employees with depressive symptoms with a care-as-usual control group. To our knowledge, this is the first study in the field of depression treatment for employees which has an e-mental health approach that is aimed at reducing depressive symptoms and preventing sick leave. If proven effective, the results of this study could stimulate the use of web-based interventions in the worksite setting. In this study the effectiveness of the web-based guided self-help course will be studied in the workplace, but the intervention could also be applied in general healthcare.

The described study design has several limitations. First, the study population will consist of white collar workers; therefore, our results have limited external validity. However, it is difficult to develop an intervention which is applicable to all occupational groups because of variations in occupations and specific job demands. In this study we have chosen to focus on white collar workers and we expect to adjust the intervention for other occupational groups, such as nursing staff, if this current study population yields positive results.

Second, we will try to recruit subjects via different recruitment methods and companies but it is probable that we do not reach all employees with depressive symptoms. In addition, some employees with depressive symptoms may hesitate to participate in this study because of doubts about confidentiality and anonymous participation regarding their manager or employer. Due to an economic recession during the inclusion period of this study (2011–2012) job security is low. Hence, employees may be more anxious about losing their job in case their manager or employer will find out about their depressive symptoms and participation in this study. We will try to minimize this potential selection-bias by placing a clear explanation about the privacy and confidentiality of the employee in the information leaflet.

Despite these limitations, this study also has several strengths. One of these strengths concerns the economic evaluation which will be performed both from a societal and a company’s perspective. The main advantage of the societal perspective is that all costs and consequences (regardless of who pays or receives) will be taken into consideration. However, the results of the economic evaluation from the societal perspective are not directly interpretable for company decisions because they may include certain costs and consequences that are not relevant from the company’s point of view [75]. For example, resource use and costs of alternative health care is relevant from a societal perspective, but not from a company’s perspective. Therefore, it is also important to perform an economic evaluation from the company’s perspective. Furthermore, in the economic evaluation from the company’s perspective we can calculate the cost-benefits in use of the health care services from the company.

Another important strength of this study concerns the methodology. We will use a diagnostic interview at baseline and follow-up assessment. Using a diagnostic interview gives us the opportunity to describe percentages of participants meeting the criteria of a depressive disorder. It will also make our study comparable with other studies. Another methodological strength concerns the different options of methods that we will have to measure work absenteeism. Since a golden standard for collecting sick leave data does not exist, using both self-report and objective sick leave data from a database gives us the opportunity to use the advantages of both methods [75] which may lead to more accurate sick leave data. However, it may be difficult to compare the sick leave databases of the companies due to use of different methods and the accuracy of registration. At this moment, we do not know whether it is possible to use objective sick leave data as well. The final strength of this study is the process evaluation. It will give us insight in the implementation, effectiveness, and execution of the intervention.

This study will be performed in the period from 2011 until 2013 and recruitment has started in September 2011. This will be the first study in the worksite setting that will focus especially on a web-based treatment for employees with depressive symptoms.

## Competing interests

The author(s) declare that they have no competing interests.

## Authors’ contributions

PC and WvM obtained funding for the study. All authors contributed to the design of this study. AG, AK, NW and PC contributed to the making of the intervention Happy@Work. AG drafted the manuscript. All authors contributed to the further writing of the manuscript. All authors read and approved the final manuscript.

## Pre-publication history

The pre-publication history for this paper can be accessed here:

http://www.biomedcentral.com/1471-244X/13/61/prepub
